# Construction and Characterization of an *Escherichia coli* Mutant Producing Kdo_2_-Lipid A

**DOI:** 10.3390/md12031495

**Published:** 2014-03-13

**Authors:** Jianli Wang, Wenjian Ma, Zhou Wang, Ye Li, Xiaoyuan Wang

**Affiliations:** 1 State Key Laboratory of Food Science and Technology, Jiangnan University, Wuxi 214122, China; E-Mails: jlwang88@hotmail.com (J.W.); talentmarvin@sina.com (W.M.); wangzhou0920@126.com (Z.W.); liye@jiangnan.edu.cn (Y.L.); 2 Key Laboratory of Industrial Biotechnology of Ministry of Education, School of Biotechnology, Jiangnan University, Wuxi 214122, China; 3 Synergetic Innovation Center of Food Safety and Nutrition, Jiangnan University, Wuxi 214122, China

**Keywords:** Kdo_2_-lipid A, lipopolysaccharide, *rfaD*, *Escherichia coli*, ESI/MS, membrane permeability, autoaggregation, biofilm

## Abstract

3-deoxy-d-*manno*-oct-2-ulosonic acid (Kdo)_2_-lipid A is the conserved structure domain of lipopolysaccharide found in most Gram-negative bacteria, and it is believed to stimulate the innate immune system through the TLR4/MD2 complex. Therefore, Kdo_2_-lipid A is an important stimulator for studying the mechanism of the innate immune system and for developing bacterial vaccine adjuvants. Kdo_2_-lipid A has not been chemically synthesized to date and could only be isolated from an *Escherichia coli* mutant strain, WBB06. WBB06 cells grow slowly and have to grow in the presence of tetracycline. In this study, a novel *E. coli* mutant strain, WJW00, that could synthesize Kdo_2_-lipid A was constructed by deleting the *rfaD* gene from the genome of *E. coli* W3110. The *rfaD* gene encodes ADP-l-*glycero*-d-*manno*-heptose-6-epimerase RfaD. Based on the analysis by SDS-PAGE, thin layer chromatography (TLC) and electrospray ionization mass spectrometry (ESI/MS), WJW00 could produce similar levels of Kdo_2_-lipid A to WBB06. WJW00 cells grow much better than WBB06 cells and do not need to add any antibiotics during growth. Compared with the wild-type strain, W3110, WJW00 showed increased hydrophobicity, higher cell permeability, greater autoaggregation and decreased biofilm-forming ability. Therefore, WJW00 could be a more suitable strain than WBB06 for producing Kdo_2_-lipid A and a good base strain for developing lipid A adjuvants.

## 1. Introduction

3-deoxy-d-*manno*-oct-2-ulosonic acid (Kdo)_2_-lipid A, the hydrophobic anchor of lipopolysaccharide (LPS) that fixes the molecule in the outer membrane of most Gram-negative bacteria [1,2,3], is an essential cell surface component for cell survival under normal growth conditions [1,4]. LPS could initiate an innate immune response through the Toll-like receptor 4/MD2 complex on the surface of many immune cells [5,6,7,8], but the actual binding group of LPS is Kdo_2_-lipid A [6,9,10]. LPS molecules are usually large-sized, have micro-heterogeneity and are difficult for detection and quantification after uptake by cultured macrophages or injection into animals [11]; in contrast, Kdo_2_-lipid A molecules are small, have micro-homogeneity and can be easily quantified by electrospray ionization mass spectrometry (ESI/MS) [12]. The changes in lipid biochemistry associated with the stimulation of RAW 264.7 cells by Kdo_2_-Lipid A could also be elucidated with C60-SIMS and LC-MS [9]. Therefore, Kdo_2_-lipid A is a better stimulator than LPS when studying innate immune systems and has been applied to many studies [9,11,13,14]. However, Kdo_2_-lipid A has not to date been synthesized chemically, and bacteria that naturally synthesize Kdo_2_-lipid A do not exist. It is necessary to construct bacterial strains that could directly synthesize Kdo_2_-lipid A.

**Figure 1:**
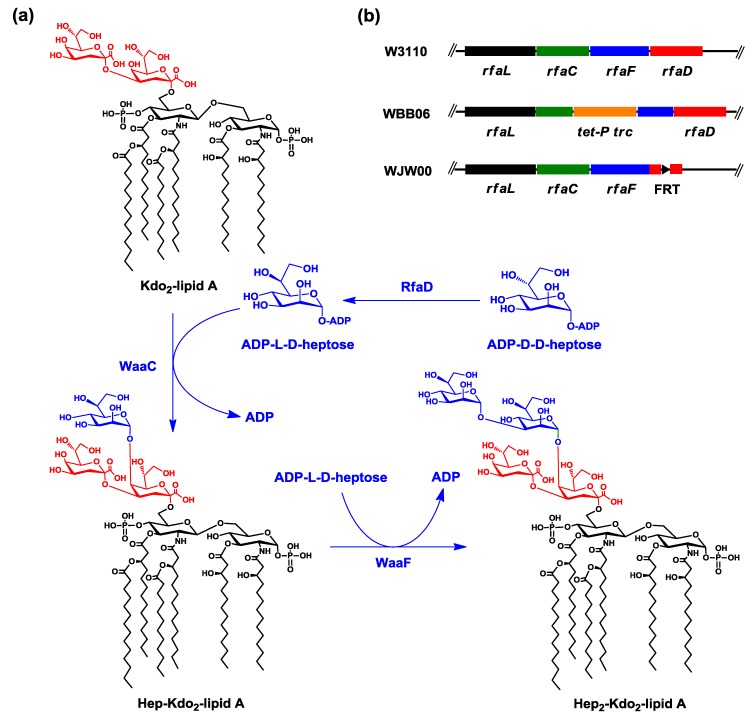
(**a**) Biosynthetic reactions from 3-deoxy-d-*manno*-oct-2-ulosonic acid (Kdo)_2_-lipid A to Hep_2_-Kdo_2_-lipid A. WaaC adds an l-d-heptose to Kdo_2_-lipid A, forming Hep-Kdo_2_-lipid A, and WaaF adds another l-d-heptose to Hep-Kdo_2_-lipid A, forming Hep_2_-Kdo_2_-lipid A. The donor of l-d-heptose is ADP-l-d-heptose, which is converted from ADP-d-d-heptose by ADP-l-*glycero*-d-*manno*-heptose-6-epimerase RfaD [19]. The lipid A, Kdo and heptose groups are shown in black, red and blue, respectively; (**b**) the comparison of the location and inactivation of genes *rfaC*, *rfaF* and *rfaD* in the chromosome of *E. coli* strains W3110, WBB06 and WJW00.

Kdo_2_-lipid A is the most conserved part of LPS among most Gram-negative bacteria [4] and is usually synthesized by nine enzymes [2,15]. However, Kdo_2_-lipid A is not accumulated in the cell, because enzymes, such as WaaC (also known as RfaC) and WaaF (also known as RfaF), could catalyze reactions that consume Kdo_2_-lipid A (Figure 1a). WaaC, a heptosyltransferase, adds an l-*glycero*-d-*manno*-heptose (l-d-heptose) to the inner 3-deoxy-d-*manno*-oct-2-ulosonic acid (Kdo) residue of Kdo_2_-lipid A, forming Hep-Kdo_2_-lipid A, and WaaF, another heptosyltransferase, adds another l-d-heptose to Hep-Kdo_2_-lipid A, forming Hep_2_-Kdo_2_-lipid A [1,16,17]. *E. coli* WaaC and WaaF are strictly monofunctional and have strong specificity for the l-d-heptose residue substrate [17,18]. The donor of l-d-heptose is ADP-l-d-heptose, which is converted from ADP-d-d-heptose by enzyme ADP-l-*glycero*-d-*manno*-heptose-6-epimerase RfaD [19,20] (Figure 1a). ADP-d-d-heptose could not be efficiently consumed by WaaC [1,16,18,20,21]. Therefore, there are two ways to accumulate Kdo_2_-lipid A *in vivo*; one is inactivating the gene, *rfaC*, encoding the enzyme, WaaC, and the other is inactivating the gene, *rfaD*, encoding the enzyme, RfaD (Figure 1b).

*E. coli* strain WBB06 (*rfaC-rfaF*::*tet6*) could accumulate Kdo_2_-lipid A [22], because both *rfaC* and *rfaF* genes in WBB06 were inactivated by inserting the *tet* gene (Figure 1b). WBB06 cells grow slowly; therefore, it is not suitable for large-scale production of Kdo_2_-lipid A. In this study, we constructed another Kdo_2_-lipid A*-*producing *E. coli* mutant strain, WJW00, by deleting the *rfaD* gene from the genome of *E. coli* W3110. WJW00 cells grow much better than WBB06 cells. The accumulation of Kdo_2_-lipid A in WJW00 was confirmed by SDS-PAGE, thin layer chromatography (TLC) and electrospray ionization mass spectrometry (ESI/MS).

## 2. Results and Discussion

### 2.1. Construction of E. coli Mutant WJW00 that Produces Kdo_2_-Lipid A by Deletion of the rfaD Gene

**Figure 2:**
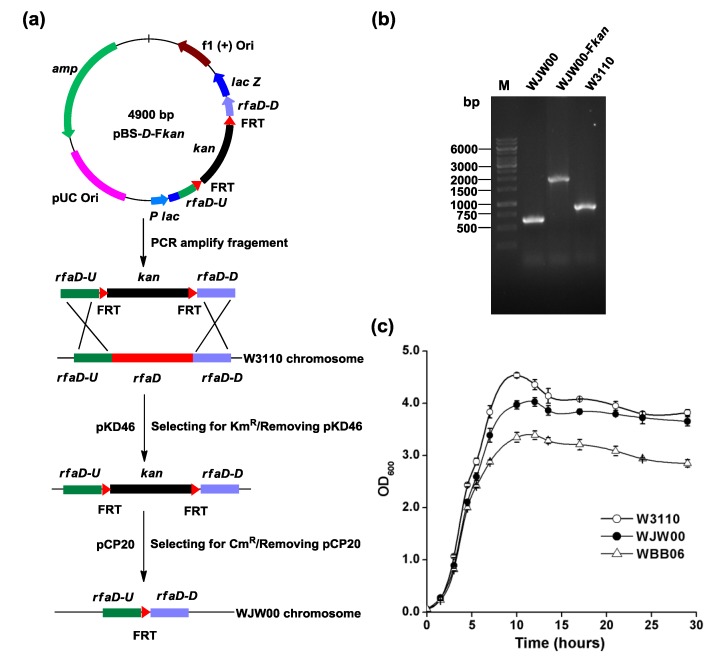
Construction of WJW00 by deleting the *rfaD* gene from the chromosome of W3110. (**a**) The *rfaD* gene from the chromosome of W3110 was replaced by the fragment, FRT-kan-FRT, with the help of pKD46. Then, the *kan* gene was deleted with the help of pCP20. Both plasmids, pKD46 and pCP20, were cured by growing at 42 °C; (**b**) the correct replacement of the *rfaD* and the later removal of *kan* were confirmed by PCR analysis. The DNA fragment sizes amplified around the region of *rfaD* in chromosomes of *E. coli* WJW00, WJW00-F*kan* and W3110 are 552, 1,854 and 889 bp, respectively; and (**c**) the growth curves of *E. coli* strains W3110, WJW00 and WBB06. All the strains were grown in LB broth at 37 °C, 200 rpm. The optical density (OD_600_) was measured at different time points. The experiment was repeated three times, and three samples were performed each time. Error bars indicate the standard deviations from three parallel samples.

To construct an *E. coli* mutant that could produce Kdo_2_-lipid A, the *rfaD* gene was deleted from the chromosome of *E. coli* W3110, as shown in Figure 2a. The plasmid, pBS-D-F*kan*, which contains a fragment, *rfaDU*-FRT-*kan*-FRT-*rfaDD*, was constructed. W3110 was first transformed with pKD46, and then with the fragment, *rfaDU*-FRT-*kan*-FRT-*rfaDD*, amplified by PCR from pBS-D-F*kan*. Red enzymes expressed by pKD46 [23] catalyzed the recombination of the FRT-*kan*-FRT cassette at the *rfaD* locus in the chromosome. The correct transformants were selected on plates containing kanamycin, and the plasmid, pKD46, was cured by growing at high temperature. Next, the plasmid, pCP20, was introduced into the cell, to allow the transient expression of FLP recombinase, which would allow the removal of the *kan* gene from the chromosome [24]. The plasmid, pCP20, was then cured by growing at high temperature. This *E. coli rfaD* mutant is designated WJW00. The correct replacement of the *rfaD* and the later removal of *kan* were confirmed by PCR analysis (Figure 2b) and kanamycin resistance. DNA fragment sizes amplified around the region of *rfaD* in chromosomes of *E. coli* WJW00, WJW00-F*kan* and W3110 are 552, 1854 and 889 bp, respectively.

Colony morphology of WJW00 was similar to wild-type W3110, but different from WBB06. Colonies of WBB06 are sticky with a smooth surface, probably due to the accumulation of l-d-heptose [17,25] and the changes of colanic acid capsule production [26,27]. The growth profiles of W3110, WJW00 and WBB06 in liquid media were different (Figure 2c). WJW00 grew slightly slower than W3110, but faster than WBB06. After 12 h, the optical density (OD_600_) of WJW00 reached 4.03, while OD_600_ of WBB06 was only 3.39. After 28 h, WJW00 was nearly equivalent to the wild-type, W3110, and 20% more cell mass was obtained from both strains than from WBB06. The defect in the biosynthesis of LPS could result in the instability of the outer membrane, associated with deficiency in FtsZ-ring formation, which affects the cell division [28]. The higher cell density and faster growth of WJW00 indicated that it is more suitable than WBB06 for the large-scale production of Kdo_2_-lipid A.

### 2.2. SDS-PAGE and TLC Analysis of Kdo_2_-Lipid A Produced by WJW00

**Figure 3:**
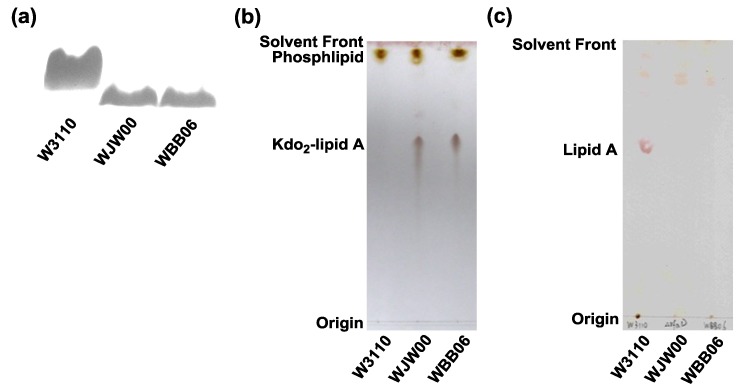
Comparison of lipids composition in *E. coli* strains W3110, WBB06 and WJW00. (**a**) The silver-stained SDS-PAGE analysis of LPS isolated from W3110, WJW00 and WBB06; (**b**) thin layer chromatography (TLC) of lipids directly extracted from W3110, WJW00 and WBB06; and (**c**) TLC of lipid A extracted from cells of W3110, WJW00 and WBB06.

Using the hot phenol/water method [29], LPSs were extracted from *E. coli* strains W3110, WBB06 and WJW00 and analyzed by SDS-PAGE [30] (Figure 3a). Clearly, LPSs extracted from WBB06 and WJW00 migrate at a similar speed, but faster than LPS extracted from the wild-type, W3110. These data suggest that the sizes of LPSs isolated from both WBB06 and WJW00 are the same and smaller than the size of LPSs from W3110. Because WBB06 produces Kdo_2_-lipid A, WJW00 might produce the same molecule.

Using the modified Bligh-Dyer method [11,15,31,32], membrane lipids were extracted from *E. coli* strains W3110, WBB06 and WJW00 and separated on TLC. The cells were extracted with a single phase Bligh-Dyer mixture. Insoluble material was collected by centrifugation, and the supernatant, containing the Kdo_2_-lipid A and glycerophospholipids, was converted to a two-phase Bligh-Dyer system. The insoluble material, containing the lipid A covalently attached to LPS, was hydrolyzed by sodium acetate and also converted to a two-phase Bligh-Dyer system. The two phases were separated by centrifugation, and the lower phases were dried by rotary evaporation. Lipids isolated from both the supernatants and the insoluble materials from all three strains were separated with TLC (Figure 3b,c). In both cases, WJW00 lipids showed the same pattern as WBB06 lipids, but different from W3110 lipids. Glycerophospholipids, the faster migrating bands, were observed in all three lipid samples from the supernatants; Kdo2-lipid A, the slower migrating bands, were observed in samples from WJW00 and WBB06, but not the W3110 sample (Figure 3b). Moreover, qualitatively repeated TLC experiments showed that a similar amount of Kdo_2_-lipid A could be produced by an equal amount cells of WJW00 and WBB06. This indicates that WJW00 produces Kdo_2_-lipid A, like WBB06, while W3110 produces LPS, which should be in the insoluble material. This is further confirmed by hydrolyzing the insoluble material and isolating lipids (Figure 3c). A typical lipid A band was observed on the TLC in samples isolated from W3110 (Figure 3c, Lane 1), but the band was not observed in samples isolated from WJW00 and WBB06 (Figure 3c, Lanes 2 and 3). This experiment suggests that all LPS in WJW00 exist as Kdo_2_-lipid A.

### 2.3. ESI/MS Analysis of Kdo_2_-Lipid A Produced by WJW00

**Figure 4:**
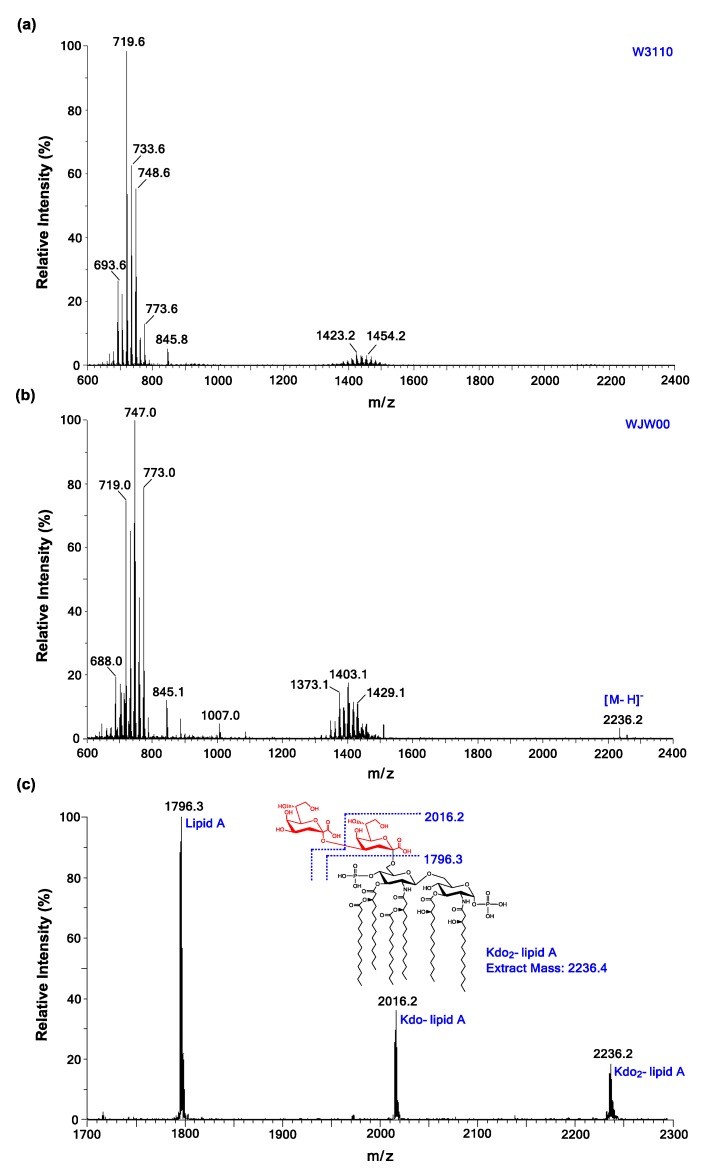
Electrospray ionization mass spectrometry (ESI/MS) and ESI/MS/MS analysis of Kdo_2_-lipid A produced by WJW00. (**a**) MS spectrum of lipids directly extracted from W3110 cells; (**b**) the MS spectrum of lipids directly extracted from WJW00 cells reveals that the peak at *m*/*z* 2236.2 is attributed to Kdo_2_-lipid A; and (**c**) ESI/MS/MS spectrum of the Kdo_2_-lipid A ion at *m*/*z* 2236.2.

The lipids directly extracted from WJW00, WBB06 and W3110 were analyzed by ESI/MS in the negative ion mode. Lipid samples from WJW00 (Figure 4b) and WBB06 showed the same MS spectra, containing the [M-H]^−^ ion peak of Kdo_2_-lipid A at *m*/*z* 2236.2 [11,12]. The peak of Kdo_2_-lipid A at *m*/*z* 2236.2 was not observed in the spectrum of lipids from wild-type W3110 (Figure 4a). The major peaks around *m*/*z* 750 (Figure 4a,b) were observed in all three spectra and should be derived from phospholipids [15,33].

To further confirm the molecular ion of Kdo_2_-lipid A at *m*/*z* 2236.2 in the spectrum of WJW00 lipids, the ion was subjected to MS/MS analysis. Except for the molecular ion at *m*/*z* 2236.2, there were two major peaks observed in the MS/MS spectrum (Figure 4c). The peaks at *m*/*z* 2016.2 and 1796.3 should be derived by the loss of one or two Kdo residues from the molecular ion, respectively.

### 2.4. The Cell Surface Properties of WJW00

**Figure 5:**
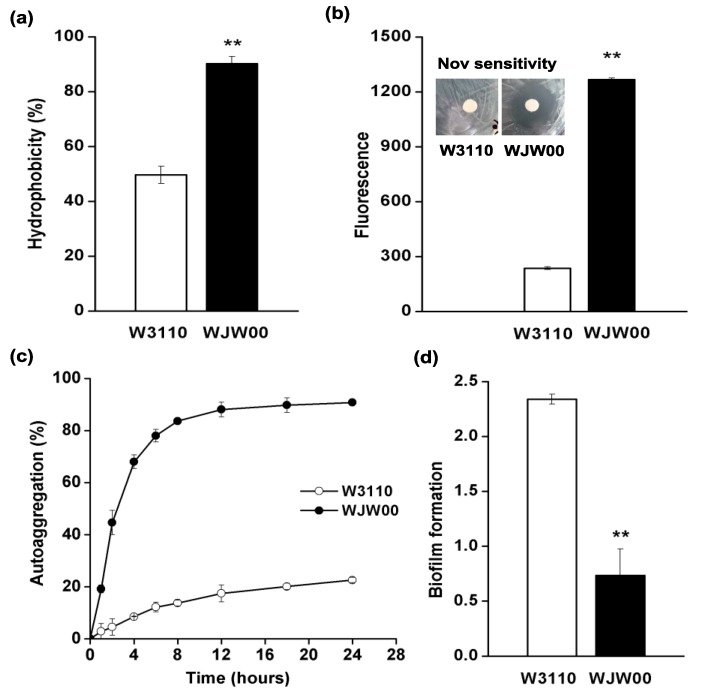
(**a**) Comparison of hydrophobicity for WJW00 and W3110 cells. Absorbance at OD_595_ in the water phase was quantified before and after 3-h incubation at room temperature; (**b**) comparison of membrane permeability for WJW00 and W3110 cells. The insert shows images of disc diffusion tests on novobiocin sensitivity for W3110 and WJW00 cells; (**c**) comparison of autoaggregation for WJW00 and W3110 cells. Absorbance at OD_595_ in the upper 0.5-cm culture was determined at different time points; (**d**) comparison of biofilm formation for WJW00 and W3110 cells. Significant differences between WJW00 and W3110 are shown in panels a, b and d. * *p* < 0.05; ** *p* < 0.01. All the experiments were repeated three times, and three samples were performed each time. Error bars indicate the standard deviations from three parallel samples.

Because a high amount of LPS molecules are distributed on the cell surface, the change of LPS to Kdo_2_-lipid A in WJW00 might change its cell surface properties [1]. Therefore, the hydrophobicity, permeability, autoaggregation and biofilm formation of WJW00 cells were studied, using the wild-type W3110 as a control. Both the hydrophobicity and permeability of WJW00 cells increased relative to W3110. The hydrophobicity of WJW00 was 1.8-fold greater than that of W3110 (Figure 5a). The membrane permeability of WJW00 was 5.3-fold greater than that of W3110 (Figure 5b). Because the polysaccharides of LPS are hydrophilic, LPS molecules provide bacteria a resistant barrier to hydrophobic antibiotics and other compounds [34]. Kdo_2_-lipid A is the hydrophobic part of LPS and is much shorter than LPS, so WJW00 cells are more hydrophobic than W3110 cells, and the outer membrane of WJW00 is more permeable than that of W3110. LPS plays an important role in membrane permeability [35]. The changed permeability may cause the decrease of outer membrane protein incorporation in WJW00 [34,36]. Since they have different membrane permeabilities, WJW00 and W3110 show different resistances to antibiotics [34]. Both disc diffusion tests and MIC measurement showed that WJW00 is more sensitive to novobiocin, a hydrophobic antibiotic. The inhibition zone of novobiocin for WJW00 is much larger than that of W3110 (Figure 5b inset). The MICs of WJW00 and W3110 to novobiocin are 8 ng/μL and 400 ng/μL, respectively.

The cell autoaggregation was also increased for WJW00. As shown in Figure 5c, after a 4-h incubation at 22 °C, the autoaggregation percentage could reach up to 70% for WJW00, but only 10% for W3110; after a 12-h incubation, 90% of WJW00 cells aggregated, but only 20% of W3110 cells aggregated (Figure 5c). In addition, WJW00 cells aggregated more easily than WBB06 cells. After centrifugation at 4000 rpm for 10 min, the OD_600_ values of the supernatants for WJW00 and WBB06 cells (the same initial OD_600_ value of 5.0) were 0.157 and 0.555, respectively. The stronger autoaggregation ability of WJW00 would benefit the large-scale production of Kdo_2_-lipid A, because the cell is easier to collect.

The biofilm formation ability of WJW00 was also studied when incubating at 37 °C without or with shaking (100 rpm). In both cases, WJW00 cells formed less biofilm than W3110. Without shaking, W3110 could form six-fold more biofilm than WJW00; with shaking, W3110 could form three-fold more biofilm than WJW00 (Figure 5d). The decreased ability for biofilm formation might be related to not only the decreased length of LPS [37,38], but also the lack of flagella [39,40] and stronger ability of autoaggregation for WJW00 [41].

Put together, the higher hydrophobicity, higher permeability, stronger ability of autoaggregation and weaker biofilm-forming ability make WJW00 highly suitable for large-scale fermentation to produce Kdo_2_-lipid A.

## 3. Experimental Section

### 3.1. DNA Preparation and PCR Techniques

Plasmid DNA was prepared using the EZ-10 spin column plasmid mini-prep kit from Bio Basic Inc. (Markham, Canada). Fragments prepared in this study were obtained by polymerase chain reaction (PCR). PCR reaction mixtures (50 μL) contained 5 μL 10× Ex Taq buffer, 4 μL dNTP mixture (2.5 mM each), 0.5 μL template (100 ng/μL), 1 μL forward primer and reverse primer (20 μM), 0.25 μL TaKaRa Ex Taq DNA polymerase and 38.75 μL ddH_2_O. The PCR reaction was first heated to 95 °C and maintained for 5 min or 10 min, followed by a 35-cycle reaction. Each cycle included 4 steps: denaturation at 95 °C for 30 s, annealing at 55 °C for 30 s, extension at 72 °C for 2 min and incubation at 72 °C for 10 min. The resulting PCR products were purified using the TIANgel midi purification kit from Tiangen (Beijing, China). TaKaRa Ex Taq DNA polymerase, restriction enzymes, T4 DNA ligase and the DNA ladder were purchased from Sangon (Shanghai, China). Primer synthesis was performed by Sangon, and they are listed in Table 1.

**Table 1:** Primers used in this study. The recognition sites for restriction enzymes are underlined.

Primers	Sequence (5′–3′)	Restriction Site
*rfaD-*U-F	CCGCTCGAGTCCGTTACACCTTCAGCA	*Xho*I
*rfaD-*U-R	CGGAATTCGTGGCTGATGTGAATCTGTGGT	*EcoR*I
*rfaD-*D-F	CCCAAGCTTTACTTGCCGTCCCACTCG	*Hind*III
*rfaD-*D-R	AAAACTGCAGGCTTTATCGGCAGCAACA	*Pst*I
*kan*-FRT-F	CGGAATTCGTGTAGGCTGGAGCTGCTTCG	*EcoR*I
*kan*-FRT-R	CCCAAGCTTGCCATTAATTCACTGATCAG	*Hind*III

### 3.2. Construction of WJW00 and Growth Analysis

The bacterial strains and plasmids used in this study are listed in Table 2. The medium for all strains contained (g/L) glucose 10, peptone 5 and NaCl 10. In the slant medium, 17 g/L agar was added. When required, antibiotics were added at an appropriate final concentration (μg/mL), 100 for ampicillin, 30 for kanamycin, 25 for chloramphenicol and 12 for tetracycline. Strains containing the temperature-sensitive plasmid pKD46 or pCP20 were grown at 30 °C, while grown at 42 °C when required to remove them. Other strains were grown at 37 °C.

**Table 2:** Bacterial strains and plasmids used in this study.

Strains or Plasmids	Description	Source
*Strains*		
W3110	Wild-type *E. coli*, F^−^, λ^−^	Laboratory strain
W3110/pKD46	W3110 transformed by pKD46	Laboratory strain
WBB06	W3110 mutant with a mutation of *waaC* and *waaF* genes (*rfaC-rfaF*::*tet6*)	[22]
WJW00	W3110 Δ*rfaD*	This Study
*Plasmids*		
pBluscript II SK+	Cloning vector, ColE1, *lacZ*, Amp^R^	Stratagene
pBS-*D*-F*kan*	Plasmid for deleting *rfaD* in *E. coli*	This Study
pKD46	P_araB_γβ exo, Rep^ts^, Amp^R^	[23]
pKD13	oriR6K, FRT Kan^R^ FRT, Amp^R^	[23]
pCP20	FLP^+^, λ cI857, λp_R_Rep^ts^, Cam^R^, Amp^R^	[24]

The λ Red recombination [23] was used to knockout the *rfaD* gene from the W3110 genome (Figure 2). The plasmid, pBS-*D*-F*kan*, was constructed by inserting a DNA fragment, *rfaD*U-FRT-*kan*-FRT-*rfaD*D, in the vector pBlueScript II SK+. Therefore, a 1350-bp kanamycin resistance cassette flanking with FRT sites was amplified by PCR using primers *kan*-FRT-F and *kan*-FRT-R from pKD13, cleaved by *EcoR*I and *Hind*III. Then, an *rfaD*-upstream 260-bp fragment was obtained using primers *rfaD-*U-F and *rfaD-*U-R, and an *rfaD*-downstream 244-bp fragment was obtained using primers *rfaD-*D-F/*rfaD-*D-R, both from the W3110 genome. The upstream and downstream fragments were cleaved by *Xho*I/*EcoR*I and *Hind*III/*Pst*I, respectively. Together with the cleaved *kan* fragment in the middle, the two chromosomal fragments were cloned into the *Xho*I and *Pst*I sites of pBlueScript II SK+. For homologous recombination, the 1854-bp fragment containing *kan* and homologous arms was amplified by PCR with primers *rfaD-*U-F and *rfaD-*D-R from pBS-*D*-F*kan*. Then, the prepared 1854-bp fragment DNA was typically electroporated into the l-ara-induced electroporation-competent cells [23]. The first recombination mutant would be obtained with the help of Red recombination plasmid, pKD46. Then the removal of *kan* gene was done using plasmid pCP20, containing Flp, after the removal of pKD46 at 42 °C. The successful insertion and deletion of the resistance cassette was confirmed by PCR analysis and loss of resistance. The WJW00 mutant without any resistance was obtained after the removal of pCP20 at 42 °C.

The growth curves of strains incubated in LB broth without any addition of antibiotic were measured (Figure 2c) at 200 rpm, 37 °C. The seed culture inoculated from an agar plate was cultivated in a 5-mL tube. When the culture grew to OD_600_ = 3.0, the seed culture was transferred into 50-mL LB broth/250-mL flask. The initial OD_600_ of all strains was adjusted to 0.02, and absorbance at OD_600_ was measured at different time points. The colonies of all strains were incubated on an agar plate for 24 h.

### 3.3. Extraction and SDS-PAGE Analysis of LPS

The LPS of all strains was isolated using the hot phenol-water extraction method [29,42], with minor modifications. Briefly, the cell pellets were harvested from 1 mL of cultures approximately at an OD_600_ of 1.0. Then, the cell pellets were resuspended in 100 μL of TAE buffer and mixed with 200 μL of Solution I, consisting of 3% SDS, 0.6% Tris and 6.4% of 2 M NaOH. The mixture was heated at 100 °C for 15 min with gently mixing every 5 min. The LPSs in the mixture were extracted with 250 μL of phenol-chloroform (1:1, *v/v*) by centrifugation at 12,000 rpm for 5 min. The 200-μL supernatant was transferred to a new centrifuge tube and mixed with 200 μL of H_2_O and 50 μL of sodium acetate (3 M and pH 5.2). Then, 500 μL of absolute ethanol were added for removing miscellaneous proteins and then centrifuged at 12,000 rpm for 5 min. The precipitation was dissolved in 200 μL of Solution II, containing 50 mM Tris-hydrochloride (pH 8.0) and 100 mM sodium acetate, and then mixed with 400 μL of absolute ethanol. Finally, LPS samples were collected after centrifugation at 12,000 rpm for 5 min and then dissolved in 50 μL of sterilized ddH_2_O. Five microliters of each LPS sample were separated on 15% SDS-polyacrylamide gels and visualized by silver staining (Figure 3a), as described by Tsai *et al*. [30,42].

### 3.4. Isolation and TLC Analysis of Lipids

To further elucidate the lipopolysaccharide structure of WJW00, total lipids were extracted using the modified Bligh-Dyer method [11,31,32]. Samples were analyzed by TLC [42,43] and electrospray ionization mass spectrometry (ESI/MS) [42]. Typically, 400-mL cultures were grown to an OD_600_ of 1.5. The cells were harvested by centrifugation at 4000 rpm for 10 min and washed with 150 mL of 0.1 M NaCl. The cell pellets were resuspended in 16 mL of 1.0 M NaCl, and then 20 mL of chloroform and 40 mL of methanol were added to form a 76-mL single-phase mixture (chloroform/methanol/1.0 M NaCl, 1:2:0.8, *v/v/v)*. The mixture was stirred for 1.0 h at room temperature. After centrifugation at 2,000 rpm for 30 min, an LPSs-containing core exist in the debris, while most Kdo_2_-lipid A exists in the supernatant. For the supernatant containing Kdo_2_-lipid A, 20 mL of chloroform and 20 mL of 1.0 M NaCl were added to generate a two-phase system. The insoluble residues were washed twice with the single-phase mixture for the extraction of Lipid A. The pellets were then resuspended in 27 mL of 12.5 mM sodium acetate and heated at 100 °C for 30 min to release lipid A from LPS. The suspensions were also converted to two-phase Bligh-Dyer mixtures by adding 30 mL of chloroform and 30 mL of methanol. After thorough mixing, the samples were centrifuged at 2000 rpm for 30 min. The lower organic phases containing lipid A or Kdo_2_-lipid A were both recovered and dried by rotary evaporation and stored at −20 °C.

The lipid samples were separated on a silica gel 60 TLC plate. The plates spotted with total lipids containing Kdo_2_-lipid A were developed in the solvent of chloroform, methanol, acetic acid and water (25:15:4:4; *v/v/v/v*) [32,42]. The TLC plate spotted with lipid A samples was developed in the solvent of chloroform, methanol, water and ammonia (40:25:4:2 *v/v/v/v*) [43].

### 3.5. ESI/MS Analysis of Kdo_2_-Lipid A

The mass spectra were acquired on a Waters SYNAPT Q-TOF mass spectrometer equipped with an ESI source (Water Corp., Milford, MA, USA). Mass spectrometry, such as the ionization techniques of electrospray ionization, had been applied to the analysis of lipids [12]. The total lipids extracted from all strains were analyzed by ESI/MS. Lipid samples were dissolved in the solvent of chloroform and methanol (2:1, *v/v*) and immediately infused into the ion source and scanned in the negative-ion mode at 0.2 µL/min. The negative ion ESI/MS was carried out at −150 V, and the collisional activation of ions was performed at −6 V. The collisional activation of ESI/MS/MS was performed at −50 V. Data acquisition and analysis were performed using MassLynx V4.1 software (Water Corp., Milford, MA, USA).

### 3.6. Surface Hydrophobicity Assay

The surface hydrophobicity of cells was determined according to the method of Wang LQ *et al*. [42]. Briefly, cell pellets were harvested from overnight culture and washed twice with PBS, pH 7.4, then resuspended in the PBS to OD_600_ around 0.5, recorded as A_0_. The 2-mL suspension was mixed with 800 μL of xylene and then incubated at room temperature for 3 h. The OD_600_ of the aqueous phase after extraction with xylene was recorded as A, and the value of [(A_0_ − A)/A_0_] × 100 represents the hydrophobicity of the bacterial cells [42].

### 3.7. Outer Membrane Permeability and Novobiocin Sensitivity Assay

Outer membrane permeability was measured by the fluorescence absorption of cells with *N*-phenyl naphthylamine (NPN) [44]. The harvested cells were washed and resuspended in 20 mM PBS, pH 7.4. Then, the 1.92-mL suspensions, adjusted to an OD_600_ of 0.5 with the above PBS, were quickly mixed with 80 μL of NPN (1 mM). The fluorescences of the mixtures were immediately monitored by a spectrofluorometer (Hitachi, Tokyo, Japan). The excitation wavelength of 350, the emission wavelength of 420 and a slit of 7 nm were used for the experiment. The fluorescence absorption per OD_600_ value indicated the cells’ outer membrane permeability.

Moreover, novobiocin sensitivity was analyzed by an MIC test and an antibiotic inhibition test filter [2,45]. For MIC tests, bacteria were diluted to 96-well culture dishes to OD_600_ = 0.02. Gradually increased novobiocin concentrations (µg/mL) of 2, 4, 8, 16, 32, 64, 100, 200, 300 and 400 in culture dishes were used to measure MIC for strains, with fresh LB broth as the control. They were incubated at 37 °C for 2 days. The MICs of bacteria were determined by the minimum concentration of novobiocin to inhibit cell growth. Antimicrobial susceptibility tests were further analyzed by disk diffusion [45]. WJW00 and W3110 were grown to the late log phase and diluted into LB broth to an OD_600_ of 0.02. A lawn of cells was spread onto a LB broth agar plate. Sterile filter paper disks (6 mm in diameter) were placed on the top of the lawn, and 10 µg (10 µL of a 1 mg/mL stock) of novobiocin were spotted onto each disk. Plates were incubated overnight (18 to 19 h) at 37 °C, and zone diameters around disks were measured to assess the novobiocin sensitivity.

### 3.8. Autoaggregation Assay

The cell autoaggregation ability was determined by the modified method as described by Wang, *et al*. [42,46]. Briefly, cells harvested from overnight culture were resuspended in the fresh liquid LB and adjusted to an OD_600_ around 2.5. The initial OD_600_ was recorded as A_0_. With the 10-mL resuspensions, the tubes were incubated without shaking at 22 °C for 24 h. The values of OD_600_ of the upper 0.5-mL suspensions after incubation for 1 h, 2 h, 4 h, 6 h, 8 h, 12 h, 18 h and 24 h were recorded as A_i_. The autoaggregation ability was expressed as the autoaggregation percentage, and the value of [(A_0_ − A_i_)/A_0_] × 100 represents the autoaggregation ability [42,46].

### 3.9. Biofilm Assay

The biofilm assay was performed in plastic tubes with a conical bottom [42]. All the OD_600_ of the 2-mL cultures were adjusted to 0.02, and three tubes with 2-mL fresh LB broth were used as the control. The tubes without shaking and with shaking at 100 rpm were both incubated at 37 °C for 3 days. To measure the biofilm formation, the cultures were poured out slightly, and the tubes were washed twice with ddH_2_O. Next, to the tubes were added 3 mL of 0.1% crystal violet, and they remained at room temperature for 10 min. After removal of the crystal violet, the tubes were washed twice with ddH_2_O. Then, 2 mL of 33% acetic acid were added in the tubes to dissolve the stained biofilm [42]. The OD_595_ of the mixture was measured and represented the biofilm formation.

For all the experiments for phenotype analysis, data were all expressed as the means ± standard deviation and all experiments were carried out in triplicate. The means were compared using the least significant difference test. *p* < 0.05 was considered a significant difference, and *p* < 0.01 was considered an extremely significant difference. All data were analyzed with SPSS Statistics 17.0 (SPSS Inc., Chicago, IL, USA) [43].

## 4. Conclusions

Kdo_2_-lipid A stimulates the innate immune system through the TLR4/MD2 complex [6]. With many advantages, Kdo_2_-lipid A became an important stimulator for studying the mechanism of the innate immune system and for developing bacterial vaccine adjuvant [11]. Recent studies on the minimal structures of LPS in *E. coli* and various lipid A structure modifications showed that Kdo_2_-lipid A is a better stimulator than lipid A or the complete LPS [32,47,48,49,50,51].

However, Kdo_2_-lipid A is not readily chemically synthesized. The *E. coli* mutant, WBB06, could be used for producing Kdo_2_-lipid A, but it grows slowly and contains an antibiotic resistant marker *(tet)* on its chromosome. The *E. coli* mutant, WJW00, we constructed here not only synthesizes Kdo_2_-lipid A, but also grows better than WBB06 and contains no antibiotic resistance genes in its chromosome. Therefore, WJW00 would be a novel *E. coli* strain suitable for the large-scale production of Kdo_2_-lipid A. WJW00 could also be used as a base strain for developing lipid A adjuvants.

## References

[1] Raetz C.R., Whitfield C. (2002). Lipopolysaccharide endotoxins. Annu. Rev. Biochem..

[2] Wang X., Quinn P.J. (2010). Lipopolysaccharide: Biosynthetic pathway and structure modification. Progr. Lipid Res..

[3] Wang X., Quinn P.J. (2010). Endotoxins: Lipopolysaccharides of Gram-Negative Bacteria. Endotoxins: Structure, Function and Recognition.

[4] Opiyo S.O., Pardy R.L., Moriyama H., Moriyama E.N. (2010). Evolution of the Kdo_2_-lipid A biosynthesis in bacteria. BMC Evol. Biol..

[5] Beutler B. (2004). Inferences, questions and possibilities in Toll-like receptor signalling. Nature.

[6] Beutler B., Rietschel E.T. (2003). Innate immune sensing and its roots: the story of endotoxin. Nat. Rev. Immunol..

[7] Miller S.I., Ernst R.K., Bader M.W. (2005). LPS, TLR4 and infectious disease diversity. Nat. Rev. Microbiol..

[8] Akira S., Uematsu S., Takeuchi O. (2006). Pathogen recognition and innate immunity. Cell.

[9] Passarelli M.K., Ewing A.G., Winograd N. (2013). C(60)-SIMS studies of glycerophospholipid in a LIPID MAPS model system: KDO(2)-Lipid A stimulated RAW 264.7 cells. Surf. Interface Anal..

[10] Srivastava H.C., Breuninger E., Creech H.J., Adams G.A. (1962). Preparation and properties of polysaccharide-lipid complexes from *Serratia marcescens*. Can. J. Biochem. Physiol..

[11] Raetz C.R., Garrett T.A., Reynolds C.M., Shaw W.A., Moore J.D., Smith D.C., Ribeiro A.A., Murphy R.C., Ulevitch R.J., Fearns C. (2006). Kdo_2_-Lipid A of *Escherichia coli*, a defined endotoxin that activates macrophages via TLR-4. J. Lipid Res..

[12] Murphy R.C., Raetz C.R., Reynolds C.M., Barkley R.M. (2005). Mass spectrometry advances in lipidomica: collision-induced decomposition of Kdo_2_-lipid A. Prostaglandins Other Lipid Mediat..

[13] Sims K., Haynes C.A., Kelly S., Allegood J.C., Wang E., Momin A., Leipelt M., Reichart D., Glass C.K., Sullards M.C. (2010). Kdo_2_-lipid A, a TLR4-specific agonist, induces de novo sphingolipid biosynthesis in RAW264.7 macrophages, which is essential for induction of autophagy. J. Biol. Chem..

[14] Kim E.Y., Shin H.Y., Kim J.Y., Kim D.G., Choi Y.M., Kwon H.K., Rhee D.K., Kim Y.S., Choi S. (2010). ATF3 plays a key role in Kdo_2_-lipid A-induced TLR4-dependent gene expression via NF-kappaB activation. PLoS One.

[15] Reynolds C.M., Raetz C.R. (2009). Replacement of lipopolysaccharide with free lipid A molecules in *Escherichia coli* mutants lacking all core sugars. Biochemistry.

[16] Kadrmas J.L., Raetz C.R. (1998). Enzymatic synthesis of lipopolysaccharide in *Escherichia coli*. Purification and properties of heptosyltransferase I. J. Biol. Chem..

[17] Gronow S., Brabetz W., Brade H. (2000). Comparative functional characterization *in vitro* of heptosyltransferase I (WaaC) and II (WaaF) from *Escherichia coli*. Eur. J. Biochem..

[18] Grizot S., Salem M., Vongsouthi V., Durand L., Moreau F., Dohi H., Vincent S., Escaich S., Ducruix A. (2006). Structure of the *Escherichia coli* heptosyltransferase WaaC: Binary complexes with ADP and ADP-2-deoxy-2-fluoro heptose. J. Mol. Biol..

[19] Coleman W.G. (1983). The *rfaD* gene codes for ADP-l-*glycero*-d-*manno*heptose-6-epimerase. An enzyme required for lipopolysaccharide core biosynthesis. J. Biol. Chem..

[20] Deacon A.M., Ni Y.S., Coleman W.G., Ealick S.E. (2000). The crystal structure of ADP-l-*glycero*-d-*manno*heptose 6-epimerase: Catalysis with a twist. Structure.

[21] Chang P.C., Wang C.J., You C.K., Kao M.C. (2011). Effects of a HP0859 (*rfaD*) knockout mutation on lipopolysaccharide structure of *Helicobacter pylori* 26695 and the bacterial adhesion on AGS cells. Biochem. Biophys. Res. Commun..

[22] Brabetz W., Muller-Loennies S., Holst O., Brade H. (1997). Deletion of the heptosyltransferase genes *rfaC* and *rfaF* in *Escherichia coli* K-12 results in an Re-type lipopolysaccharide with a high degree of 2-aminoethanol phosphate substitution. Eur. J. Biochem..

[23] Datsenko K.A., Wanner B.L. (2000). One-step inactivation of chromosomal genes in *Escherichia coli* K-12 using PCR products. Proc. Natl. Acad. Sci. USA.

[24] Cherepanov P.P., Wackernagel W. (1995). Gene disruption in *Escherichia coli*: TcR and KmR cassettes with the option of Flp-catalyzed excision of the antibiotic-resistance determinant. Gene.

[25] Ding L., Seto B.L., Ahmed S.A., Coleman W.G. (1994). Purification and properties of the *Escherichia coli* K-12 NAD-dependent nucleotide diphosphosugar epimerase, ADP-l-*glycero*-d-*manno*heptose 6-epimerase. J. Biol. Chem..

[26] Kanipes M.I., Papp-Szabo E., Guerry P., Monteiro M.A. (2006). Mutation of *waaC*, encoding heptosyltransferase I in *Campylobacter jejuni* 81–176, affects the structure of both lipooligosaccharide and capsular carbohydrate. J. Bacteriol..

[27] Parker C.T., Kloser A.W., Schnaitman C.A., Stein M.A., Gottesman S., Gibson B.W. (1992). Role of the *rfaG* and *rfaP* genes in determining the lipopolysaccharide core structure and cell-surface properties of *Escherichia-coli* K-12. J. Bacteriol..

[28] Fujishima H., Nishimura A., Wachi M., Takagi H., Hirasawa T., Teraoka H., Nishimori K., Kawabata T., Nishikawa K., Nagai K. (2002). *kdsA* mutations affect FtsZ-ring formation in *Escherichia coli* K-12. Microbiology.

[29] Kido N., Ohta M., Kato N. (1990). Detection of lipopolysaccharides by ethidium bromide staining after sodium dodecyl sulfate-polyacrylamide gel electrophoresis. J. Bacteriol..

[30] Tsai C.M., Frasch C.E. (1982). A sensitive silver stain for detecting lipopolysaccharides in polyacrylamide gels. Anal. Biochem..

[31] Bligh E.G., Dyer W.J. (1959). A rapid method of total lipid extraction and purification. Can. J. Biochem. Physiol..

[32] Zhao J., Raetz C.R. (2010). A two-component Kdo hydrolase in the inner membrane of *Francisella novicida*. Mol. Microbiol..

[33] Wang X., Ribeiro A.A., Guan Z., Raetz C.R. (2009). Identification of undecaprenyl phosphate-beta-d-galactosamine in *Francisella novicida* and its function in lipid A modification. Biochemistry.

[34] Delcour A.H. (2009). Outer membrane permeability and antibiotic resistance. Biochim. Biophys. Acta.

[35] Nikaido H. (2003). Molecular basis of bacterial outer membrane permeability revisited. Microbiol. Mol. Biol. Rev..

[36] Vaara M. (1992). Agents that increase the permeability of the outer-membrane. Microbiol. Rev..

[37] Bandara H.M., Lam O.L., Watt R.M., Jin L.J., Samaranayake L.P. (2010). Bacterial lipopolysaccharides variably modulate *in vitro* biofilm formation of *Candida* species. J. Med. Microbiol..

[38] Lau P.C., Lindhout T., Beveridge T.J., Dutcher J.R., Lam J.S. (2009). Differential lipopolysaccharide core capping leads to quantitative and correlated modifications of mechanical and structural properties in *Pseudomonas aeruginosa* biofilms. J. Bacteriol..

[39] Beloin C., Roux A., Ghigo J.M. (2008). *Escherichia coli* biofilms. Curr. Top. Microbiol. Immunol..

[40] Wood T.K., Gonzalez Barrios A.F., Herzberg M., Lee J. (2006). Motility influences biofilm architecture in *Escherichia coli*. Appl. Microbiol. Biotechnol..

[41] Schembri M.A., Kjaergaard K., Klemm P. (2003). Global gene expression in *Escherichia coli* biofilms. Mol. Microbiol..

[42] Wang L., Hu X., Tao G., Wang X. (2012). Outer membrane defect and stronger biofilm formation caused by inactivation of a gene encoding for heptosyltransferase I in *Cronobacter sakazakii* ATCC BAA-894. J. Appl. Microbiol..

[43] Han Y., Li Y., Chen J., Tan Y., Guan F., Wang X. (2013). Construction of monophosphoryl lipid A producing *Escherichia coli* mutants and comparison of immuno-stimulatory activities of their lipopolysaccharides. Mar. Drugs.

[44] Vaara M. (1990). Antimicrobial susceptibility of *Salmonella typhimurium* carrying the outer membrane permeability mutation SS-B. Antimicrob. Agents Chemother..

[45] Wang X., Ribeiro A.A., Guan Z., Abraham S.N., Raetz C.R. (2007). Attenuated virulence of a *Francisella* mutant lacking the lipid A 4′-phosphatase. Proc. Natl. Acad. Sci. USA.

[46] Rahman M., Kim W.S., Kumura H., Shimazaki K. (2008). *In vitro* effects of bovine lactoferrin on autoaggregation ability and surface hydrophobicity of *bifidobacteria*. Anaerobe.

[47] Raetz C.R., Reynolds C.M., Trent M.S., Bishop R.E. (2007). Lipid A modification systems in gram-negative bacteria. Annu. Rev. Biochem..

[48] Needham B.D., Carroll S.M., Giles D.K., Georgiou G., Whiteley M., Trent M.S. (2013). Modulating the innate immune response by combinatorial engineering of endotoxin. Proc. Natl. Acad. Sci. USA.

[49] Vorachek-Warren M.K., Ramirez S., Cotter R.J., Raetz C.R. (2002). A triple mutant of *Escherichia coli* lacking secondary acyl chains on lipid A. J. Biol. Chem..

[50] Klein G., Lindner B., Brabetz W., Brade H., Raina S. (2009). *Escherichia coli* K-12 suppressor-free mutants lacking early glycosyltransferases and late acyltransferases: Minimal lipopolysaccharide structure and induction of envelope stress response. J. Biol. Chem..

[51] Wang X., Karbarz M.J., McGrath S.C., Cotter R.J., Raetz C.R. (2004). MsbA transporter-dependent lipid A 1-dephosphorylation on the periplasmic surface of the inner membrane: topography of *Francisella novicida* LpxE expressed in *Escherichia coli*. J. Biol. Chem..

